# Metastatic breast cancer mimicking a hilar cholangiocarcinoma: case report and review of the literature

**DOI:** 10.1186/1477-7819-12-384

**Published:** 2014-12-16

**Authors:** Martina Coletta, Roberto Montalti, Mirco Pistelli, Paolo Vincenzi, Federico Mocchegiani, Marco Vivarelli

**Affiliations:** Department of Gastroenterology and Transplantation, Hepatobiliary and Abdominal Transplantation Surgery, Marche Polytechnic University, AOU Ospedali Riuniti, via Conca 71, 60129 Ancona, Italy; Department of Medicine, Oncology Clinic, Marche Polytechnic University, AOU Ospedali Riuniti, via Conca 71, 60129 Ancona, Italy

**Keywords:** Bile duct tumors, Breast cancer, Breast cancer metastases

## Abstract

**Background/aims:**

Breast cancer is the most common tumor in women and the first cause of death for malignancy in the female population. Bile ducts are not among the common sites of metastasis from breast cancer; few cases of obstructive jaundice due to metastatic breast cancer have been described in the literature and they mostly resulted from widespread liver metastases that eventually involved the bile ducts. We report an exceptional case of metastatic infiltration of the extrahepatic bile ducts in absence of liver metastases.

**Case presentation:**

A 56-year-old woman who had undergone a right mastectomy 13 years earlier due to infiltrating ductal breast cancer and had remained tumor free, presented at a follow-up examination with obstructive jaundice.

Imaging (computed tomography, magnetic resonance and endoscopic retrograde cholangiopancreatography) scans showed features that were suggestive of a primary tumor of the extrahepatic bile duct. At surgery, the intraoperative findings were also those of a tumor of the bile duct, however, an histological examination showed no evidence of malignancy in the mucosa, but did shown an infiltration of the external wall from adenocarcinoma. Immunohistochemistry analysis demonstrated that the tumor was metastatic breast cancer.

**Conclusions:**

Indeterminate stenosis of the extrahepatic bile ducts should be examined with suspicion in women with a history of breast cancer, and bile duct metastases are to be considered among the possible diagnoses. A differential diagnosis from cholangiocarcinoma is of paramount importance and mainly relies on pathology.

## Background

Strictures of the proximal tract of the extrahepatic bile ducts are caused by hilar cholangiocellular carcinoma in over 80% of cases. Other possible causes of proximal biliary obstruction are inflammatory and stone disease, sclerosing cholangitis or, more rarely, spread from gallbladder cancer [1].

Despite all improvements and new developments in available diagnostic tools, a significant proportion of bile duct stenoses remain indeterminate before surgery. While it is reported that 13 to 24% of the presumed diagnoses of hilar cholangiocarcinoma are not confirmed at surgery, where they are found to be benign [2], the possibility of a malignancy other than cholangiocellular carcinoma is not generally taken into account.

Breast cancer is the most common malignancy in women, accounting for 29% of all female tumors [3]; it is the main cause of death from cancer in females and the second in the general population, after lung cancer [4, 5]. In 10% of the cases distant metastases are already present at the time of the diagnosis [6]. Breast cancer metastases occur through contiguous, lymphatic and hematogenous spread. Common sites of metastasis include bone, lung, lymph nodes, liver and brain [7]. Widespread liver metastases that compress or infiltrate the bile ducts can sometimes cause obstructive jaundice, whilst a direct metastatic involvement of the extrahepatic bile ducts in absence of hepatic lesions is exceptional.

We report a case of obstructive jaundice due to a metastatic deposit from breast cancer on the outer layer of the extrahepatic bile ducts that mimicked a hilar cholangiocellular carcinoma. The literature was reviewed to ascertain whether such a case had ever been reported in the past.

## Case presentation

**Figure 1:**
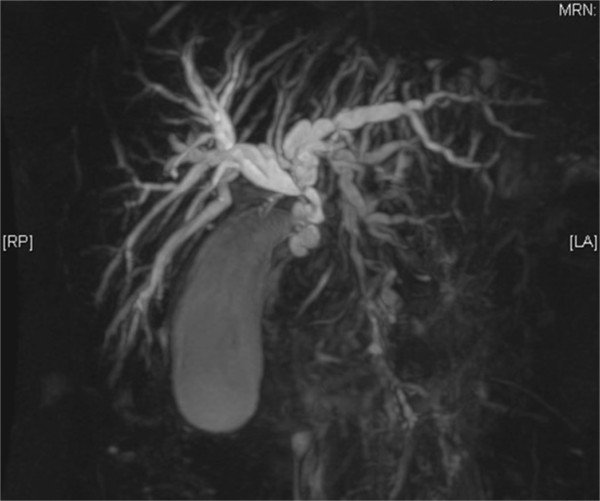
**Magnetic resonance imaging showing the stenosis of the middle portion of the common bile duct and the dilatation of intra- and extra-hepatic biliary tree.**

In February 2013 a 56-year-old female was referred to our institution due to obstructive jaundice. Laboratory tests showed increased aspartate aminotransferase (AST) and alanine aminotransferase (ALT) serum levels (206 UI/ml and 656 UI/ml, respectively), and total and conjugated hyperbilirubinemia levels (9.5 mg/dl and 8.1 mg/dl, respectively). Tumor markers, namely carcinoembryonic antigen (CEA), cancer antigen 19-9 (CA 19-9), alpha-fetoprotein (AFP), cancer antigen15-3 (CA 15-3) and cancer antigen 125 (CA 125) were within normal ranges. An abdominal ultrasound (US) and computed tomography (CT) scan showed severe non-lithiasic hydropic gallbladder with intra- and extrahepatic dilatation of the biliary tree and thickening of the common bile duct wall. There was no evidence of growth in the head of pancreas, liver or peribiliary tissue. Magnetic resonance imaging (MRI) showed a 2-cm stenosis of the middle portion of the common bile duct, while the distal tract was apparently disease-free (Figure 1).

Endoscopic retrograde cholangiopancreatography (ERCP) confirmed the presence of marked dilatation of the biliary tree that started from the proximal portion of the common bile duct and involved the confluence of the hepatic ducts. Cytology of the brushing from the bile duct showed no malignant cells.

In the patient’s past medical history there was a right radical mastectomy with axillary lymph node dissection for breast cancer performed 13 years earlier. At that time there was no evidence of distant metastasis. Pathology of the primary breast cancer revealed a 2-cm invasive ductal AJCC grade 2 carcinoma. Nipple, surgical borders and all of the 16 lymph nodes removed were free from malignancy. Essays for hormonal receptors were weakly positive, with estrogen receptors at 4.2 fmol/ml and progesterone receptors at 1.5 fmol/ml; a human epidermal growth factor receptor 2 (HER2)/Ki-67 test was not performed. After a mastectomy, the patient received adjuvant chemotherapy based on six cycles of CMF regimen (cyclophosphamide 600 mg/m^2^, methotrexate 40 mg/m^2^, 5-fluorouracil 600 mg/m^2^ intravenously on days one to eight, every four weeks), and was treated for the following three years with adjuvant hormonal therapy based on luteinizing hormone-releasing hormone (LH-RH) antagonist (triptorelin) only. Regular oncological follow-up were instituted and no recurrence had been detected through the years.

**Figure 2:**
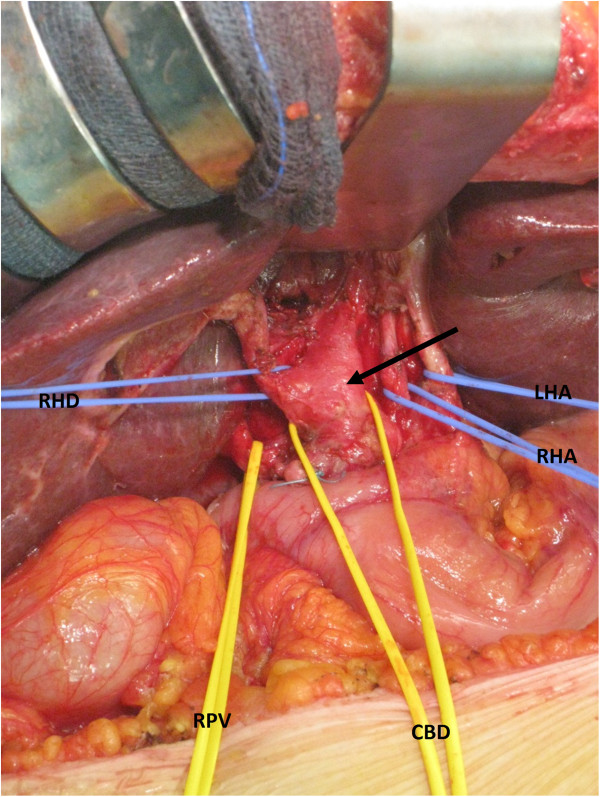
**Intraoperative image: isolation of the biliary and vascular elements of the hepatic hilum.** Note the thickened wall of the common (arrow) and right/left bile ducts. CBD: common bile duct; LHA: left hepatic artery; RHA: right hepatic artery, RHD: right hepatic duct; RPV: right branch of the portal vein.

As the presumed diagnosis was that of primary bile duct tumor, in March 2013, the patient underwent a laparotomy (Figure 2). In addition to the mentioned abdominal imaging, the preoperative work-up included a chest X-ray that showed no lesion.

At surgery, there was no evidence of peritoneal or hepatic tumor spread. A retropancreatic lymph node was removed and sent for extemporaneous histological examination which showed the presence of metastatic adenocarcinoma; it was not possible to define the origin of the adenocarcinoma on the frozen section. The bile duct was thickened and of hard consistency at palpation up to the confluence of the main hepatic ducts. After a cholecystectomy, the whole extrahepatic biliary tract was resected, including both right and left ducts. An extemporaneous histological examination of the resected biliary ducts showed an absence of malignancy in the mucosa, while there was infiltration of the external wall from the adenocarcinoma, with cells disposed in honeycombs. Because of the undetermined origin of the malignancy and its wide extension, it was decided to avoid any hepatic or pancreatic resection and the bile drainage was restored through a double hepaticojejunostomy. The patient’s postoperative course was uneventful, with rapid return of normal liver function, and the patient was discharged after seven days.

**Figure 3:**
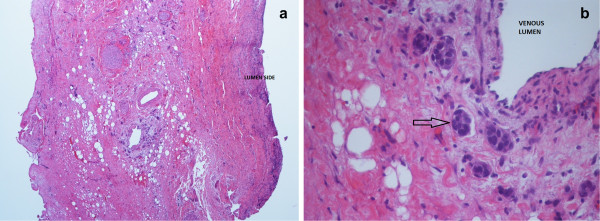
**Hematoxylin and eosin stain from an optical microscope at low magnification (40×, a) and high magnification (400×, b) of the bile duct wall.** In **(a)**, the whole thickness of the wall is included in the view field; as specified in the image, the lumen of the bile duct is on the right side of the histological specimen, and it is covered by normal mucosa. In **(b)** there is a detail of the external side of the bile duct wall: perivascular and intravascular (arrow) invasion of metastatic cells are shown.

The definitive pathological study described solid honeycombs of malignant epithelial cells, localised only in the external side of the biliary duct wall, while the mucosa was free from infiltration (Figure 3). Several images of perivascular and perineural invasion were described. Lymph nodes were extensively metastatic. An immunohistochemistry analysis of the tumor in the bile duct and the lymph nodes showed high proliferative activity (Mib-1 < 32%); cells were strongly positive for estrogen receptors (90%) and also positive for progesterone receptors (20%), mammaglobin and cytokeratin-7 (CK-7). Staining was negative for HER2/neu and cytokeratin-20 (CK-20) markers. These findings were in keeping with hormone receptor positive, HER2/neu negative metastatic breast cancer.

The patient was referred to an oncologist who decided on a treatment course of denosumab (120 mg subcutaneously every four weeks) and letrozole (2.5 mg per day), which are still ongoing and well tolerated. At present she is alive and well and there is no evidence of relapse in the liver or bile ducts.

## Conclusions

In the presence of a bile duct stricture at the hepatic hilum it can be difficult to establish a diagnosis prior to surgery, even when several investigations such as serum tumor markers, cholangiole MRI, ERCP with intraductal brushing, endoscopic ultrasound and, where available, cholangioscopy are combined. On the other hand, the fact that cholangiocellular carcinoma is the most common cause of those strictures justifies a surgical approach, whereas a malignancy cannot be excluded.

Virtually, every site of the human body can be targeted by hematogenous spread of breast cancer, however, metastases to the digestive tract, the kidneys and retroperitoneal organs have been only occasionally reported. Metastatic breast cancer can cause obstructive jaundice when multiple liver lesions are present, the bile duct is compressed by enlarged lymph nodes or, more rarely, when the head of the pancreas is targeted. The biliary tract is very rarely affected by metastases in general, and in these cases, colorectal cancer is the most frequent malignancy involved [8]. Isolated breast cancer metastasis to the biliary tract, gallbladder and Vater ampulla are exceptional. By reviewing the literature, we were able to find only 27 cases of extrahepatic biliary tract metastasis from breast cancer (Table 1) [9–19]. Of these 27 cases, four were reported in publications that were not available in full (Feliu Villaro *et al*. [10] whose article is only available in Spanish, and Rabin and Richter [16] as the journal is not available in the Italian library system.

**Table 1:** **Reported cases of metastatic breast cancer involving the bile ducts**

Author	Number of cases	Reported localisation	Treatment
Popp *et al*. 1979 [15]	7	Extrahepatic lymph nodes, wall of the common bile duct (1 case)	Radical and palliative surgery, transhepatic drainage, radiation, chemotherapy
Rabin and Richter *et al.* 1979 [16]	3	Undetermined	Undetermined
Kopelson *et al*. 1980 [12]	6	Extrahepatic duct	Radical and palliative surgery, radiation, chemotherapy
Engel *et al*. 1980 [9]	2	Extrahepatic duct, lymph nodes	Biliary tract resection and choledochojejunostomy
Franco *et al*. 1987 [11]	2	Proximal bile duct, bifurcation.	Bile duct resection, double choledochojejunostomy, cholecystectomy
Pappo *et al*. 1991 [14]	1	Extrahepatic, intra and extraluminal	Bile duct resection, choledochojejunostomy, cholecystectomy
Feliu Villaró *et al*. 1995 [10]	1	Extrahepatic intraluminal	Undetermined
Papo *et al*. 1996 [13]	1	Extrahepatic intraluminal	Biliary tract resection and choledochojejunostomy
Titus *et al*. 1997 [19]	1	Distal bile duct	Pancreaticoduodenectomy
Stoeckler *et al*. 2007 [18]	1	Distal bile duct	Pancreaticoduodenectomy
Rego *et al*. 2009 [17]	2	Ampulla of Vater, intraluminal	Surgery, observation

In the largest series reported (reported in the 1970s), Popp *et al*. [15] described seven cases of women who underwent surgery to relieve a biliary obstruction from metastatic breast cancer at a median time of 40 months from the diagnosis of the primary tumor. The pre-operative work-up was mainly based on ERCP and percutaneous transhepatic cholangiography (PTC) as CT and MRI scans were not available at those times, therefore the diagnosis of metastatic involvement of the bile duct was considered unlikely before surgery. At surgery, two patients had liver metastases and, in six of the seven cases, bile duct compression from lymph-node metastases was the cause of the obstruction. Eventually only one patient had direct metastasis to the bile duct [15]. In the second largest series from Kopelson *et al*. (also reported in the 1970s), the extrahepatic biliary system was reported as the ‘initial site’ metastasis, however, no further details were provided [12].

In an article published by Engel *et al*. in 1980, two cases were described; the bile duct was compressed by an intraperitoneal mass in one case and by enlarged metastatic lymph nodes in the other [9]. [11] reported two cases of metastases of the proximal bile duct in women whose breast cancer had been diagnosed six and eight years earlier. In the first of these cases the tumor was mainly located in the lymphatic ducts within the bile duct wall while in the other it was transmural [11]. Differently from the case herein described, in the reports from Pappo *et al*. [14] and Papo *et al*. [13] the tumor involved the lumen of the bile duct, while in the cases described by Titus *et al*. [19] and Stoeckler *et al*. [18] it originated from the Ampulla of Vater and infiltrated the bile duct wall.

A permanent feature of all the reports available in the literature is that the diagnosis was not achieved nor suspected before surgery, with the only exception being Rego *et al*. who reported two cases where the bile duct was involved in the periampullary area and the endoscopic biopsy allowed a preoperative diagnosis [17]. Similarly to what we observed, in the majority of the cases there was a long interval between the diagnosis of the primary tumor and the development of metastases affecting the biliary tract; this timing can make it hard to suspect a relationship between the breast cancer and the biliary disease.

As far as we know, the present case is unique as only the external side of the bile ducts was involved by the tumor, which made it impossible to reach a diagnosis before the specimen was removed. However, the absence of malignancy in the mucosal side of the bile ducts and the peculiar disposition of the tumor cells in external wall were not in keeping with the diagnosis of cholangiocellular carcinoma, and therefore contraindicated more aggressive surgery involving hepatic resection.

The present report provides some evidence that metastases from breast cancer can target the extrahepatic bile ducts in the absence of liver involvement, and the invasion of the bile duct wall can also take place without evidence of disease in the lumen or the mucosa of the duct, thus making the preoperative diagnosis particularly difficult. In patients with a history of breast cancer, the possibility of a biliary localisation of metastatic disease should be considered in the differential diagnosis of obstructive jaundice due to a bile duct stenosis whose origin is unclear. In similar circumstances the first goal that can be achieved is to rule out the diagnosis of cholangiocellular carcinoma to avoid unnecessary demolition of the hepatic parenchyma. At surgery, frozen sections should be obtained and the pathologist should be informed of the medical history of breast cancer. In cases where the histological features are not typical of cholangiocellular carcinoma, hepatic resection should be avoided.

## Consent

Written informed consent was obtained from the patient for publication of this Case report and any accompanying images. A copy of the written consent is available for review by the Editor-in-Chief of this journal.

## Abbreviations

AFP: alpha-fetoprotein
ALT: alanine aminotransferase
AST: aspartate aminotransferase
CA19-9: cancer antigen
CA15-3: cancer antigen
CA 125: cancer antigen
CEA: carcinoembryonic antigen
CK-7: CK-20: cytokeratin-7-20
CT: computed tomography
ERCP: endoscopic retrograde cholangiopancreatography
HER2: human epidermal growth factor receptor 2
LH-RH: luteinizing hormone releasing hormone
MRI: magnetic resonance imaging
PTC: percutaneous trans hepatic cholangiography
US: ultrasound.
